# Muscle activity in asymmetric bench press among resistance-trained individuals

**DOI:** 10.1007/s00421-020-04476-5

**Published:** 2020-08-27

**Authors:** Atle Hole Saeterbakken, Tom Erik J. Solstad, David G. Behm, Nicolay Stien, Matthew Peter Shaw, Helene Pedersen, Vidar Andersen

**Affiliations:** 1 Department of Sport, Food and Natural Sciences, Faculty of Education, Arts and Sports, Western Norway University of Applied Sciences, Campus Sogndal, PB 133, 6851 Sogndal, Norway; 2 School of Human Kinetics and Recreation, Memorial University of Newfoundland, St. John’s Newfoundland, Canada

**Keywords:** Rehabilitation, Instability, Electromyographic, Asymmetry loading, Resistance training, Performance

## Abstract

**Purpose:**

To determine the effects of asymmetric loads on muscle activity with the bench press.

**Method:**

Seventeen resistance-trained men performed one familiarization session including testing one repetition maximum (1RM) and three 5 repetition maximum (RM) lifts; using symmetric loads, 5% asymmetric loads, and 10% asymmetric loads. The asymmetric loading (i.e., reduced load on one side) was calculated as 5% and 10% of the subject`s 1RM load. In the experimental session, the three conditions of 5RM were conducted with electromyographic activity from the pectoralis major, triceps brachii, biceps brachii, anterior deltoid, posterior deltoid, and external oblique on both sides of the body.

**Results:**

On the loaded side, asymmetric loads reduced triceps brachii activation compared to symmetric loads, whereas the other muscles demonstrated similar muscle activity between the three conditions. On the de-loaded side, 10% asymmetry in loading resulted in lower pectoralis major, anterior deltoid, and biceps brachii activation compared to 5% asymmetric and symmetric loading. On the de-loaded side, only pectoralis major demonstrated lower muscle activation than symmetric loads. Furthermore, asymmetric loads increased external oblique activation on both sides compared to symmetric loads.

**Conclusions:**

Asymmetric bench press loads reduced chest and shoulder muscle activity on the de-loaded side while maintaining the muscle activity for the loaded side. The authors recommend resistance-trained participants struggling with strength imbalances between sides, or activities require asymmetric force generation (i.e., alpine skiing or martial arts), to implement asymmetric training as a supplement to the traditional resistance training.

## Introduction

Bench press is one of the most frequently used exercises to improve upper body strength and power among athletes, fitness, and health enthusiasts. Bench press is typically performed lying supine with the head, shoulders, and buttocks in contact with the bench. The barbell is lowered to the chest before being pressed upwards until the elbows are fully extended. Due to a multiplicity of reasons, many individuals experience different strength levels between the sides of the body (asymmetry) (Schmid et al. 2010). Over time, the lack of symmetry in muscle strength between sides may result in shoulder pain, injuries, or the inability to return to sports or activities. To the authors’ knowledge, no previous study has examined the effects of performing unbalanced loads referred to as asymmetric lifting (i.e., more loads on one side of the barbell).

In rehabilitation from serious long-lasting injuries with immobility (i.e., anterior cruciate ligament ruptures or shoulder dislocation), the strength relationship between limbs is one way of measuring the progression of a rehabilitation process (Norte et al. 2020; Johnson et al. 2018). However, unequal strength between limbs may increase injury rate and reduce performance in activities or sports where symmetry in strength between limbs may be crucial (i.e., alpine skiing, sprinting) (Maloney 2019). In bench press, an imbalance of strength on one side may cause individuals to move the barbell`s center of mass more laterally towards the stronger side or, that the barbell is not lifted horizontally (i.e., one of the arms are more extended than the other) in the ascending phase. Over time, this may cause greater differences in strength between sides and, in worst case, cause an overload of the dominant side.

Unbalanced loads, like asymmetric loads, induce an unstable environment to the shoulder joints. The most common approaches to increase the stability requirement in the shoulder girdle are: to (1) replace a stable with an unstable surface (Anderson and Behm 2004; Saeterbakken and Fimland 2013b; Goodman et al. 2008), (2) use unilateral instead of bilateral exercises (Saeterbakken and Fimland 2012; Saeterbakken et al. 2015; Behm et al. 2005), or (3) increase the stability requirement in the exercises using unstable loads (i.e., free weights instead of training machines) (Welsch et al. 2005; Saeterbakken et al. 2011; Kohler et al. 2010) or unbalanced loads (Calatayud et al. 2015; Glass et al. 2016; Langford et al. 2007). Unstable loads (i.e., water filed tubes, two independent dumbbells, hanging loads in elastic bands, or asymmetric loads) have received far less attention than other approaches (Kohler et al. 2010; Langford et al. 2007; Glass et al. 2016).

With the chest-press, the use of two independent dumbbells or unilateral chest-press exercises may improve the imbalance of strength between the limbs. However, performing chest-press with dumbbells differs from the bench press in kinematics, strength, and muscle activation (Welsch et al. 2005; Saeterbakken et al. 2011; Tillaar and Saeterbakken 2012). Furthermore, unilateral, isoinertial (dynamic) chest-press with loads used to gain strength (< 6RM) has previously been proven difficult to conduct (Santana et al. 2007). Recently, loads (kettlebells and weight plates) have been attached to the barbell using elastic bands to create unstable loads, as the barbell is lowered and lifted, among powerlifters. The augmented loads resulted in similar muscle activity observed between stable loads and unstable conditions with only increased biceps brachii activity with increased stability requirements (Lawrence et al. 2018).

Using unbalanced loads in the barbell bench press may mimic the bench press movement to a greater extent than dumbbell chest-press or unilateral chest-press exercises. Furthermore, asymmetric lifting may cause a greater overload of the weak side, and over time, the imbalance in strength may be counterbalanced between sides. To the authors’ knowledge, only one previous study has examined the muscle activity between loaded and de-loaded sides in the barbell bench press using asymmetric loads (Jarosz et al. 2020). Jarosz et al. (2020) reported higher muscle activity in pectoralis major, triceps brachii, and anterior deltoid on the loaded side during 2.5%, 5.0%, and 7.5% asymmetric loading using 70% of 1RM compared to the de-loaded side, but only the anterior deltoid demonstrated consistency across conditions. However, the study was limited by not controlling the lateral movement of the barbell, meaning the participants could move the barbell lateral to counteract the asymmetric loads. Furthermore, the same absolute rather than relative intensity (i.e., 70% of 1RM in each of the conditions) was used between conditions which may explain the findings (Alkner et al. 2000; McBride et al. 2010). The aim of this study was, therefore, to compare the neuromuscular effects of lifting with the same relative intensity (5RM) using asymmetric loads (5% and 10% of 1RM) with symmetrically balanced loads in the bench press. We hypothesized similar muscle activity on the loaded side, but lower prime muscle activity (pectoralis major, triceps brachii, and anterior deltoid) with increasing asymmetry on the de-loaded side (Alkner et al. 2000; McBride et al. 2010).

## Methods

### Participants

Seventeen (17) resistance-trained men (age 26.4 ± 5.5 years, weight 81.8 ± 8.0 kg, and height 179.8 ± 6.3 cm) with 9.4 ± 4.7 years of resistance training experience were recruited. All participants were familiar with the barbell bench press and the participants’ relative strength (1RM in bench press/body weight) was 1.36 ± 0.14. None of the participants were power or weightlifters. Inclusion criteria to participate were the ability to lift their own body weight in bench press, familiar with the exercise, and free of pain which could affect maximal effort.

All participants were informed orally, and in writing, of the procedures and each gave their written consent to participate before being enrolled in the study. The participants could withdraw from the study at any time without giving a reason. The study was approved by the Norwegian Center of Research Data (959065), conformed with the University College`s ethical guidelines and the standards of treatment of human participants in the research outlined in the 5th Declaration of Helsinki.

### Study design

This study was a cross-over design where each participant attended one familiarization session determining one repetition maximum (RM) in barbell bench press. Five percent (5%) and 10% of the 1RM load were calculated for each participant and used as difference in loads between the dominant and non-dominant sides during the asymmetric lifting procedures. In the same familiarization session as the 1RM lifting, the participants lifted 5RM in bench press with 0% (i.e., symmetric), 5% and 10% reduced load on non-dominate side (i.e., asymmetry loads) in a randomized and counterbalanced order. Three-to-five days after the familiarization session, the experimental session was conducted. In the experimental session, 5RM was examined in the three conditions (i.e., 0%, 5%, and 10% asymmetry) with electromyographic (EMG) measurements of the triceps brachii, biceps brachii, anterior deltoid, pectoralis major, and external oblique on both sides of the body.

### Measurements and test procedures

Before each session, a standardized warm-up was conducted. The warm-up contained 20, 12, 6, and 2 repetitions using 20%, 50%, 70%, and 85% of the participant’s 1RM in the barbell bench press. In the familiarization session, the participant`s self-reported 1RM was used to calculate the warm-up loads, while the tested 1RM was used to calculate the warm-up loads in the experimental session. Between 2–3 min recovery was permitted between each warm-up set. The participants used their preferred foot and grip width, which were measured and controlled before each test. In the 1RM test and the three 5RM tests, the barbell had to touch the chest lightly before being elevated until the elbows were fully extended. The head, shoulders, and buttocks had to be in contact with bench at all time. Since the loads were lifted with maximal effort, the lifting speed was self-selected to improve the ecological validity. If a participant managed to complete a lift with the correct technique, the loads were increased with 1.0–5.0 kg until failure. Four-to-five minutes separated each 1RM and 5RM attempts.

**Fig. 1:**
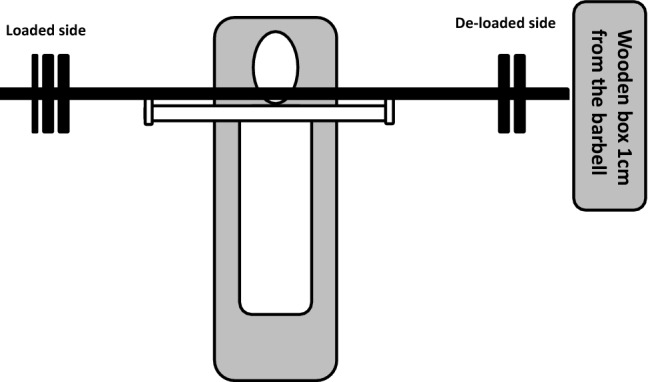
The test set-up

Five percent (5%) and 10% of the 1RM load was calculated and used in the asymmetric lifting. Meaning, if a participant lifted 100 kg with the 1RM, the loads were reduced by 5 kg (5%) and 10 kg (10%) on the non-dominant side. The preferred arm to throw a ball was defined as the dominant side. The 5% and 10% extra loads were adjusted to the closest 0.5 kg. The average asymmetric loads were 5.56 ± 0.81 kg (5%) and 11.18 ± 1.66 kg (10%). To avoid excessive lateral movement of the barbell, the backside of a wooden box was moved 1 cm from the barbell end on the de-loaded side (see Fig. 1). The participants were instructed in the familiarization session to avoid sliding the barbell against the wooden box. In addition, a test leader controlled that the center of the barbell touches the center of sternum. The coefficient of variation between the familiarization and experimental sessions for the 0%, 5%, and 10% asymmetric 5RM lifts was 0.981 (symmetric), 0.946 (5%), and 0.969 (10%).

### Surface electromyography (EMG)

Surface EMG was recorded using a Musclelab Data synchronize Unit (Musclelab 6000 system) and analyzed by the Musclelab software (Ergotest Technology AS, Porsgrunn, Norway). Before placing the electrodes, the skin was shaved, abraded, and washed with alcohol according to the previous recommendations (Hermens et al. 2000). Gel-coated self-adhesive electrodes (Dri-Stick Silver circular sEMG Electrodes AE-131, NeuroDyne Medical, USA) with an 11-mm contact diameter and a 2-cm center-to-center distance were used. The electrodes were placed using anatomical landmarks informed by SENIAM and previous studies on the pectoralis major, anterior deltoid, posterior deltoid, biceps brachii, triceps brachii, and external oblique on loaded and de-loaded sides (Saeterbakken and Fimland 2013b; Saeterbakken et al. 2016; Behm et al. 2005). To minimize noise from external sources, the raw EMG signal was amplified and filtered using a pre-amplifier (input impedance; 1000GΩ) located close to the sampling point. The common mode of rejection ratio of the pre-amplifier was 106 dB with a fourth-order Butterworth band-pass filter (high-cut frequency of 500 Hz and low-cut frequency of 20 Hz). The EMG signals were sampled at a rate of 1000 Hz and were rectified, integrated, and converted to root-mean-square (RMS) EMG signals using a hardware circuit network (frequency response 450 kHz, averaging constant 12 ms, and total error ± 0.5%). The mean RMS EMG signal of each muscle during the five repetitions of the lift was used in further analyses. Finally, the RMS EMG signals were normalized to the participants’ 5 s of maximal isometric voluntary contraction (MVC) according to the recommendations (Hermens et al. 2000) and previous studies (Saeterbakken et al. 2019). Two MVC trials were conducted for each muscle and separated by 1–2 min. The 3 s with the greatest mean RMS EMG signals were used as the subject`s MVC. More detailed information of the MVCs has been published elsewhere (Saeterbakken et al. 2019; Comfort et al. 2012; Hamlyn et al. 2007).

To identify the beginning and end of each test, a linear encoder (ET-Enc-02, Ergotest Innovation A/S, Porsgrunn, Norway) was placed underneath the barbell. The linear encoder had a resolution of 0.075 mm and counts the pulses with 0.01-s intervals and was synchronized with the EMG measurements using the Musclelab Data synchronize Unit (Ergotest Innovation A/S, Porsgrunn, Norway). The barbell velocity was calculated using a five-point differential filter with the commercial software v10.4 (Ergotest Innovation A/S, Porsgrunn, Norway).

### Statistics

The statistical analyses were conducted using SPSS statistical software (25.0, SPSS Inc., Chicago, IL, USA). To examine the differences in muscle activity between the three conditions, a repeated analysis of variance (ANOVA) for each muscle on both sides with Bonferroni post hoc tests was conducted. Repeated ANOVA was also used to examine differences in lifting time and 5RM loads in the three conditions. The data are presented as mean ± 95% confidence intervals and with Cohen’s *d* effect size (ES). An ES of < 0.2 was consider trivial, 0.2–0.5 small, 0.5–0.8 medium, and > 0.8 large (Cohen 1988). The significant level was set to > 0.05.

## Results

### Pectoralis major

**Fig. 2:**
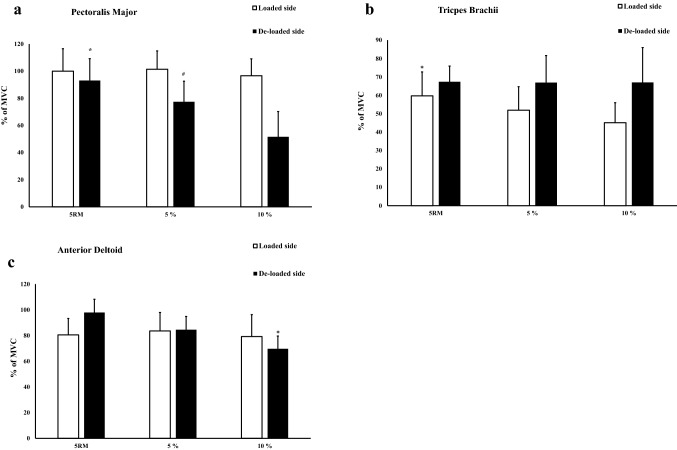
**a**–**c** The muscle activity (% of MVC) with 95% confidence interval on loaded and de-loaded sides. *Significant different (*p* < 0.05) compared to other conditions. ^#^Significant different (*p* < 0.05) compared to 10% asymmetry

On the loaded side, similar muscle activity between the three conditions (0%, 5%, and 10% asymmetry in load) was observed (*p* = 0.440–1.000, ES = 0.01–0.04, Fig. 2a). On the de-loaded side, the pectoralis major demonstrated 20.3% and 80.4% greater muscle activity with symmetric loading compared to 5% (*p* = 0.002, ES = 0.54) and 10% (*p* < 0.001, ES = 1.27) asymmetric loads (Fig. 2a).

### Triceps brachii

On the loaded side, 15.0% and 32.4% greater muscle activity was observed with symmetric loads compared to 5% (*p* = 0.013, ES = 0.32) and 10% (*p* < 0.001, ES = 0.65) asymmetric loads, respectively (Fig. 2b). No difference was observed between 5 and 10% asymmetric loads (*p* = 0.071, ES = 0.23). On the de-loaded side, similar muscle activity was observed between the three conditions (*p* = 1.000, ES = 0.04–0.25; Fig. 2b).

### Anterior deltoid

On the loaded side, similar muscle activity between the three conditions was observed (*p* = 0.490–1.000, ES = 0.11–0.24: Fig. 2c). On the de-loaded side, symmetric (*p* = 0.004, ES = 1.54) and 5% asymmetric loads (*p* = 0.005, ES = 0.72) led to 15.8% and 40.0% greater muscle activation when compared to 10% asymmetric loading (Fig. 2c). No difference was observed between symmetric and 5% asymmetric loads (*p* = 0.077, ES = 0.78; Fig. 2c).

### Biceps brachii

**Fig. 3:**
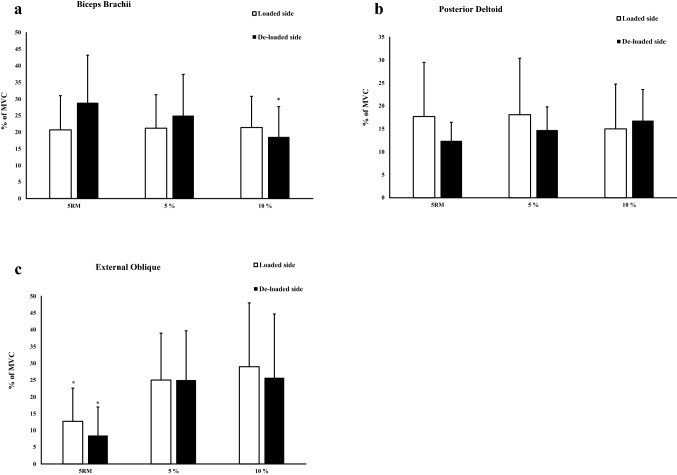
**a**–**c** The muscle activity (% of MVC) with 95% confidence interval on loaded and de-loaded side. *Significant different (*p* < 0.05) compared to other conditions

On the loaded side, similar muscle activity between the three conditions was observed (*p* = 1.000, ES = 0.00–0.05; Fig. 3a). On the de-loaded side, no difference was observed between symmetric and 5% asymmetric loads (*p* = 0.373, ES = 0.15; Fig. 3a). However, there was a 15.8% and 55.7% greater muscle activity with symmetric loads (*p* = 0.034, ES = 0.44) and 5% asymmetric loads (*p* = 0.013, ES = 0.30) compared with 10% asymmetric loads.

### Posterior deltoid

Similar muscle activity was observed between the three conditions on the loaded side (*p* = 0.166–1.000, ES = 0.04–0.18) and de-loaded side (*p* = 0.299–0.557, ES = 0.38–0.57; Fig. 3b).

### External oblique

On the loaded side, 280% and 320% greater muscle activity was observed with 5% (*p* = 0.003, ES = 1.04) and 10% (*p* < 0.001, ES = 1.35) asymmetric loads compared to symmetric loads (Fig. 3c). No difference was observed between 5 and 10% asymmetry (*p* = 1.000, ES = 0.04). On the de-loaded side, 75.0% and 133.0% greater muscle activity was observed during 5% (*p* = 0.039, ES = 0.74) and 10% (*p* = 0.004, ES = 0.99) compared to symmetric loads (Fig. 3c). No difference was observed between 5 and 10% asymmetric loads (*p* = 0.387, ES = 0.30; Fig. 3c).

There were no differences in total lifting time between the conditions (*p* = 0.206–1.000). The 5% and 10% asymmetry loads decreased by 12.2% (*p* < 0.001; ES = 0.88) and 24.6% (*p* < 0.001; ES = 1.81) compared the symmetric 5RM load (92.5 ± 13.6 kg). The 5% asymmetry loads were 16.4% greater than the 10% asymmetric loads (*p* < 0.001, ES = 0.99).

## Discussion

The main findings of the present study were that asymmetric and symmetric bench press resulted in similar muscle activation on the loaded side with the exception of greater triceps brachii and lower external oblique activation during symmetric loads. Furthermore, the de-loaded side demonstrated lower pectoralis major, anterior deltoid, and biceps brachii activation with asymmetric loads compared to symmetric loads. The 5RM loads decreased with increasing asymmetry.

As hypothesized, similar muscle activity was observed on the loaded side with the pectoralis major and anterior deltoid, whereas the muscle activity decreased with increasing asymmetry in the de-loaded side (pectoralis major only). Although the total load lifted decreased with increasing asymmetry (~ 12% and 25%), the relative intensity would have been similar (5RM) for the loaded side, but not for the de-loaded side. This explains the differences between the sides. In comparison, Jarosz et al. (2020) examined asymmetric loading (2.5%, 5.0% and 7.5%) in the right and left parts of pectoralis major and anterior deltoid in the bench press. However, the study had several methodological differences compared to the present study using the same absolute loads across the conditions, only using peak muscle activation and not controlling the lateral movement during asymmetric lifts. These limitations may explain why greater pectoralis major activity in the left (loaded) was observed compared to the right (de-loaded), but no differences were observed when the right side was loaded and compared to the left (de-loaded) side. In contrast to the present findings, greater muscle activity was observed in the anterior deltoid on the loaded side than the un-loaded side during asymmetric lifts (Jarosz et al. 2020). The previous studies examining chest-press exercises using unstable surfaces have observed similar pectoralis major activation despite decreasing loads with increased instability requirements (Saeterbakken et al. 2016; Anderson and Behm 2004; Goodman et al. 2008). However, these studies examined unstable surfaces and not unstable loading as in the present study where different loads were provided on either end of the barbell (loaded > de-loaded loads). Furthermore, the different intensities (i.e., % of 1RM) between the sides resulted in different muscle activity (Alkner et al. 2000; McBride et al. 2010; van den Tillaar et al. 2019), which explains the decreasing muscle activity in pectoralis major and anterior deltoid on the de-loaded side.

In contrast to the pectoralis major, triceps brachii demonstrated lower muscle activity on the loaded side using the two asymmetric conditions, whereas the de-loaded side demonstrated no significant difference. However, lower triceps brachii activation on the loaded side using asymmetric loads may be a result of maintaining the center of the barbell over the sternum. Using asymmetric loads, lateral forces had to be generated to compensate for the disruptive momentum caused by the de-loaded side. Triceps brachii can generate lateral forces with the bench press (Duffey and Challis 2011). The lower triceps brachii activation on the loaded side, contrasting with the similar activation across the conditions on the de-loaded side, was most likely an attempt to maintain a centered barbell. In comparison, decreased triceps activation was observed using unstable loads (i.e., dumbbells) compared to barbell chest-press (Saeterbakken et al. 2011). However, as the dumbbells’ movements are independent, high triceps activation will only extend the elbows making the lift more similar to flies with a different lifting kinematic characteristic (Saeterbakken et al. 2011; Welsch et al. 2005; Tillaar and Saeterbakken 2012).

Probably, because of the lower triceps brachii activation on the loaded side using asymmetric loads, the biceps brachii activation decreased on the de-loaded side using asymmetric loads. However, only the 10% asymmetric was lower than the two other conditions, which may be a result of greater momentum with increasing asymmetry in the loads. Furthermore, similar biceps brachii activation was observed on the loaded side between the three conditions. One of the neurological adaptions with experienced resistance training (inclusion criteria of the present study) is the inhibition of the antagonist, which explains the biceps brachii activation pattern across the conditions. The inclusion of highly experienced bench press athletes may also explain the similar anterior deltoid activation between sides and conditions. In comparison, increased stability requirement has demonstrated increased deltoid poster activation in shoulder press (Saeterbakken and Fimland 2013a; Kohler et al. 2010). However, the stability requirement may not significantly alter the posterior part of the shoulder muscle when performing asymmetric lifting which also resulted in similar muscle activation.

As hypothesized, asymmetric loading increased the external oblique activation (core muscle) on both sides. Still, no differences were observed between 5 and 10% asymmetry in the loaded side, contrasting with significant differences on the de-loaded side. With increased asymmetry, the requirement to maintain the position on the bench increases, which explains the findings. Furthermore, greater contralateral activation in the core muscle may be a result of maintaining the hip position on the bench. Similar findings have been observed in the previous studies examining increased stability requirements using unilateral instead of bilateral loads (Saeterbakken and Fimland 2012; Santana et al. 2007).

The findings of the present study are difficult to compare with the previous literature. To the authors’ knowledge, only one previous study has examined the effect of de-loading one side with the barbell bench press (Jarosz et al. 2020). However, the study had several methodological differences compared to the present which makes the results difficult to compare. The previous studies have examined instability using unstable surfaces (Saeterbakken and Fimland 2013b; Anderson and Behm 2004; Goodman et al. 2008) or unstable loads (Saeterbakken et al. 2011; Welsch et al. 2005; Santana et al. 2007; Kohler et al. 2010).

Still, the study has some strengths and limitations that need to be addressed. Only one familiarization session was conducted, and little is known of the short- or long-term effects of performing asymmetric lifting. The participants had a mean resistance training experience of approximately 10 years and excellent test–retest reliability of all conditions was observed. Furthermore, only highly resistance-trained men were recruited and the findings may not necessarily be generalized to other populations. Other populations (e.g., untrained, adolescent, elderly, or athletes) may experience force imbalances between limbs, whereas the findings may be beneficial to balance the strength levels to avoid a continued emphasis using the strongest side. There is always an inherent risk of crosstalk when examining EMG activity; however, the same experienced researcher placed all electrodes according to the previous recommendations and all data was normalized using maximal isometric voluntary contractions. Finally, to avoid excessive lateral movement to balance the loads during asymmetric loadings, the barbell lateral movement was controlled. The authors are certain that the lifts were conducted asymmetrically. Further studies should include other populations, use other repetition ranges aiming to improve strength (i.e., muscle hypertrophy and explosive strength), and examine training effects with athletes experiencing force imbalance between limbs.

## Conclusion

Asymmetric loads with the bench press reduced the muscle activity in the chest and shoulder muscles on the de-loaded side while maintaining the muscle activity for the loaded side. Asymmetric loads may be used to balance the strength between sides or used as a supplement in shoulder rehabilitation. Asymmetric training may be used in sports facing force imbalance between limbs to improve performance and to prevent injuries.

## Abbreviations

ANOVA: Analysis of variance
ES: Effect size
EMG: Electromyography
MVC: Maximum voluntary contraction
RM: Repetition maximum
RMS: Root-mean-square
